# Supplementary material for: Transcription Regulation of Cell Cycle Regulatory Genes Mediated by NtrX to Affect Sinorhizobium meliloti Cell Division
Source: Genes (Basel). 2022 Jun 15;13(6):1066. doi: 10.3390/genes13061066 (PMC9223101; doi:10.3390/genes13061066)
Supplement: Supplementary file 1 [file genes-13-01066-s001.zip › Supplementary materials 2022.pdf]

Transcription regulation of cell cycle regulatory genes mediated by NtrX to affect *Sinorhizobium meliloti* cell division

Shenghui Xing[1], Wenjia Zheng[1], Fang An[1], Leqi Huang[1], Xinwei Yang[1], Shuang Zeng[1], Ningning Li[1], Khadidja Ouenzar[1], Liangliang Yu[1], Li Luo[1*]

Supplementary materials

Table S1 Expression differentials of cell cycle regulatory genes evaluated by a RNA-seq experiment.

Table S2 DNA fragments from Sm1021 cells precipitated by anti-NtrX antibodies in a ChIP-Seq experiment.

Table S3 DNA oligonucleotides used in this study.

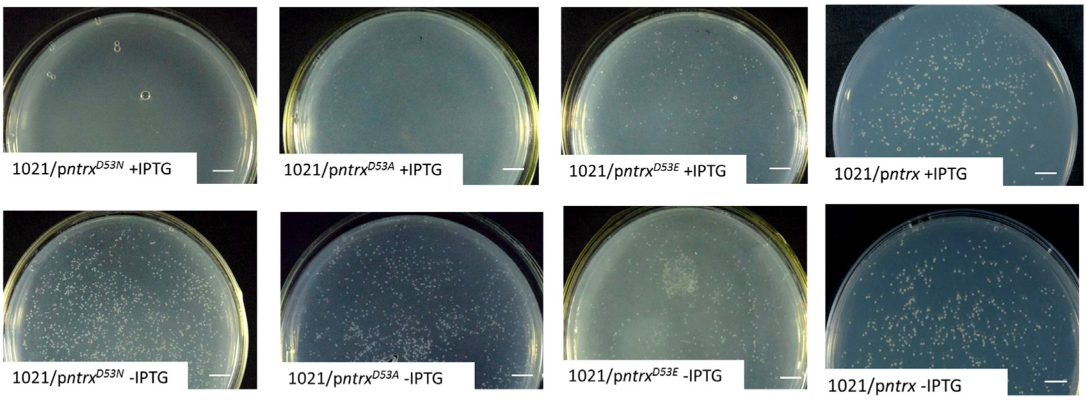

Figure S1. Colony formation of Sm1021 expressing NtrX containing a D53 substitution on LB/MC/IPTG (1mM) agar
plates.

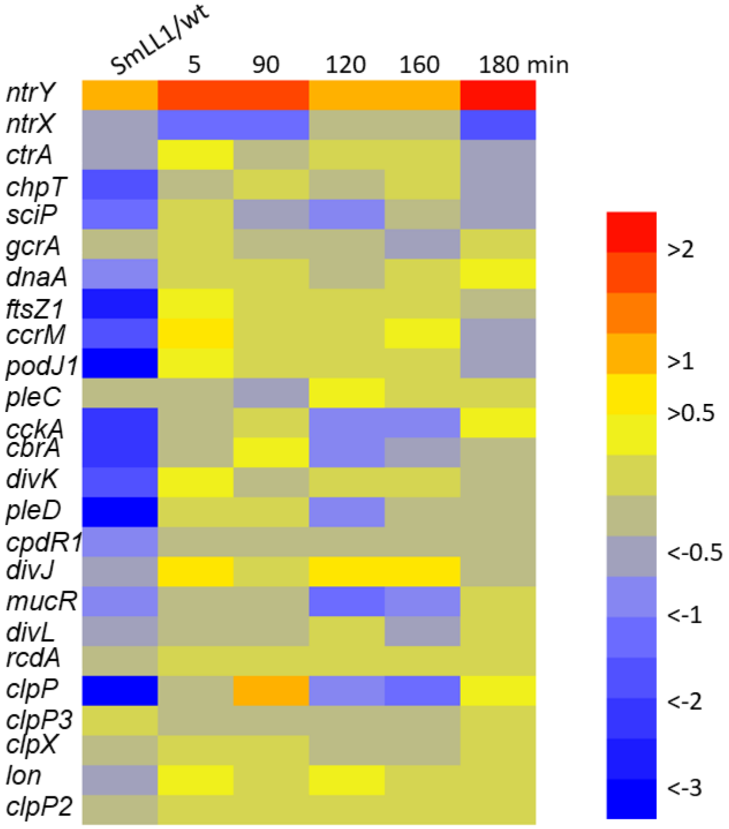

.

Figure S2. Heat map showing the expression differentials of cell cycle regulatory genes from one RNA-seq assay. The original data are listed in Table S1.

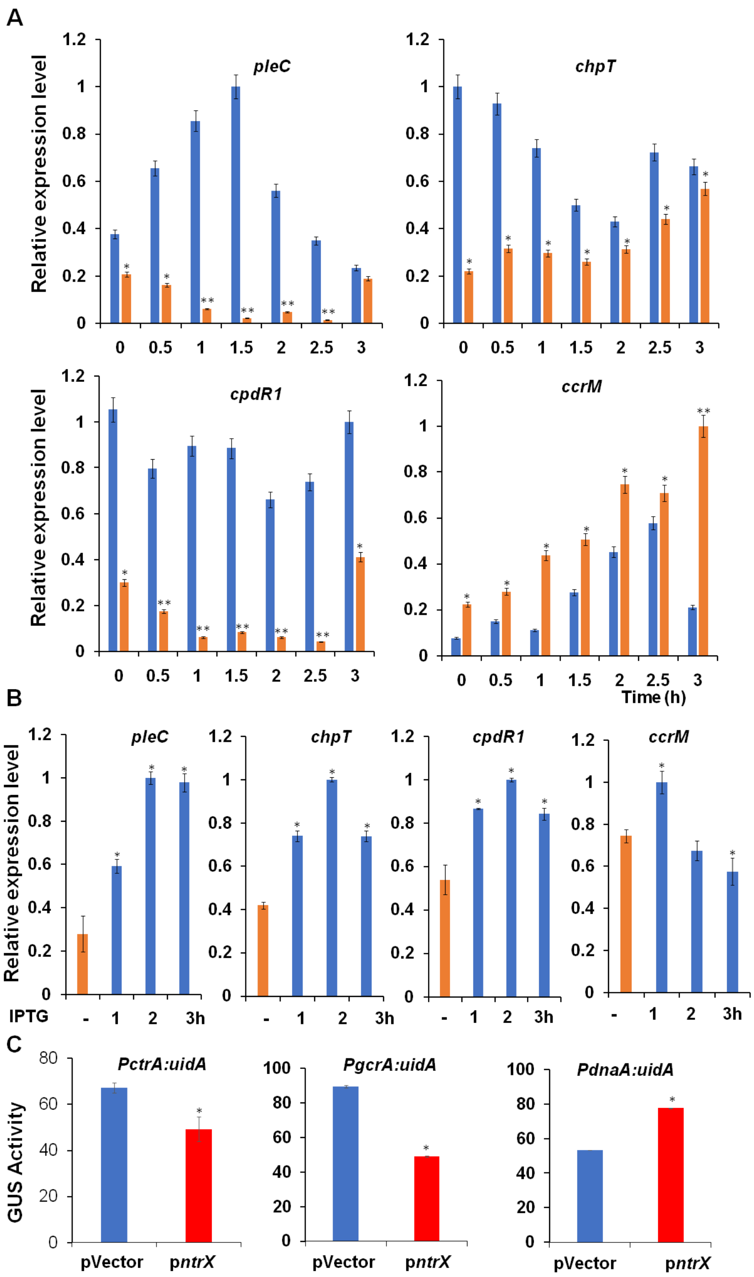
A
pleC
chpT
cpdR1
ccrM
Relative expression level
Time (h)
B
pleC
chpT
cpdR1
ccrM
Relative expression level
IPTG
- 1 2 3h
C
PctrA:uidA
PgcrA:uidA
PdnaA:uidA
GUS Activity
pVector
pntrX

Figure S3. Expression differentials of some cell cycle regulatory genes in *ntrX* mutants. (**A-B**) Transcript levels of cell cycle regulatory genes in *S. meliloti* cells were evaluated by qRT-PCR. The depletion strain was grown in LB/MC broth containing 1 mM IPTG in **B**. (**C**) GUS activities of promoter fusions of *ctrA*, *gcrA* and *dnaA* in *E. coli* DH5α cells in LB broth. Error bars, ±SD. The student t-test was used for significance analysis. *, P<0.05; **, P<0.001.

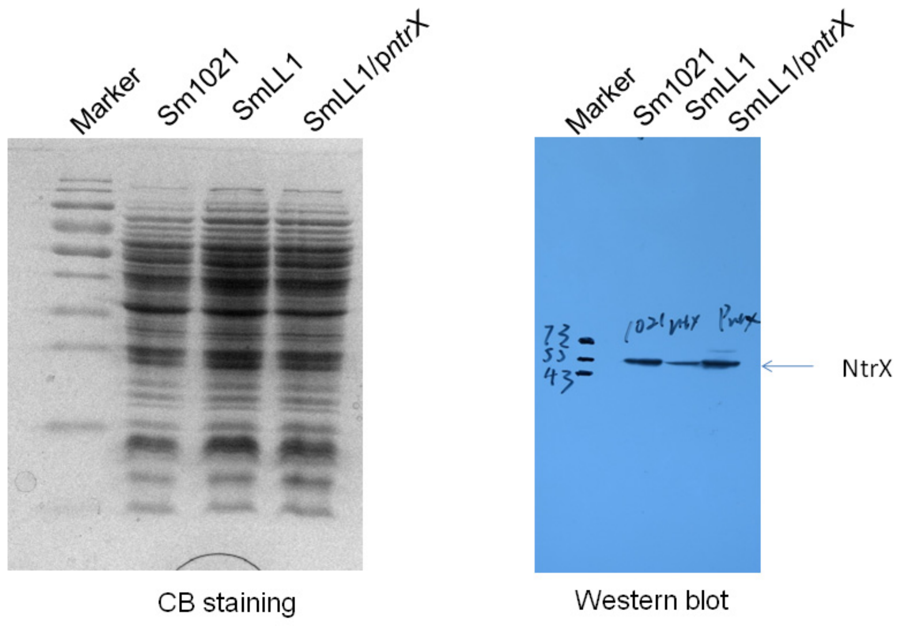

Figure S4. The NtrX protein detected by western blotting using rabbit anti-NtrX polyclonal antibodies. The antibodies were used for ChIP-seq assay.

Gene	Recognition site	Distance	TSS
cpdR1	CAAGGGACTTG	32 bp	TSS08721
dnaA	CAAAACCCTTG	455 bp	TSS00863
ntrY	CAACACCGTTG	12 bp	TSS04572
	CAA--TGCGTTG	0 bp	TSS04572
ctrA	CAA------CCTTG	0 bp	TSS0839
gcrA	CAA----GGTTTG	126 bp	TSS01427
	CAATTA---CTAG	0 bp	TSS01426
ftsZ1	CAA----TGGCTG	9 bp	TSS06915
divL	CAAC---ATCTTG	79 bp	TSS00088

Figure S5. The possible recognition sites of NtrX in promoter regions of cell cycle regulatory genes. TSS, Transcription start sites from the literature (1).

Methods and Materials

GUS activity assay

To evaluate β-glucuronidase activity, *E. coli DH5α* carrying two plasmids was grown in LB medium to OD_{600}≈0.8 at 37 °C. The cells were collected by centrifugation at 6,000 rpm for 2 min at 4°C. The cells were crushed by the electric drill (J1Z-GL-10, 220V, 50Hz, 400W, Modong Company) on ice. The cell lysate was kept on ice to analyze β-glucuronidase activity with the substrate of p-nitrophenyl-β- D-glucuronide (Sigma) as described by Jefferson (2).

RNA-Seq

RNA-seq was performed as described by An (3). Synchronized *S. meliloti* cells were subcultured in LB/MC broth containing 500 μg ml^{-1} of streptomycin at 28 °C. The cells from 5 ml of *S. meliloti* cultures were collected, RNA was isolated using Trizol Reagent (Invitrogen) and cDNA was prepared using a reverse transcription kit (TakaRa). The integrity of the RNA samples was assessed by an Agilent 2100 Bioanalyzer (Agilent). RNA-Seq was performed in Shanghai Bohao Biotechnology Corporation. The sequencing data were first preprocessed and filtered using the Seq TK method to remove small RNAs and rRNAs (https://github.com/lh3/seqtk). Genome mapping was then performed using bowtie2 (version: 2-2.0.5) for local alignment of clean reads, which is suitable for prokaryotic transcriptome sequencing data. Finally, the number of reads was converted to FPKM (fragments per kilobase of exon model per million mapped reads) to standardize gene expression. The FPKM number for each gene was counted using the HTSeq bowtie2 method and then normalized with TMM (trimmed mean of M values), and each gene was then assessed using a perl script.

References

1. Schlüter JP, Reinkensmeier J, Barnett MJ, Lang C, Krol E, Giegerich R, Long SR,
Becker A. 2013. Global mapping of transcription start sites and promoter motifs in the symbiotic alpha-proteobacterium Sinorhizobium meliloti 1021. BMC Genomics 14:156.

2. Jefferson RA, Burgess SM, Hirsh D. 1986. beta-Glucuronidase from *Escherichia coli* as a gene-fusion marker. Proc Natl Acad Sci U S A 83: 8447-8451.
3. An F, Li N, Zhang L, Zheng W, Xing S, Tang G, Yan J, Yu L, Luo L. 2021. Identification of *Sinorhizobium meliloti* LsrB regulon. Acta Biochim Biophys Sin (Shanghai). 53(7):955-957.
